# Toxicity of Pekinenin C from Euphorbia Pekinensis Radix on Rat Small Intestinal Crypt Epithelial Cell and Its Apoptotic Mechanism

**DOI:** 10.3390/ijms17060850

**Published:** 2016-06-02

**Authors:** Yudan Cao, Fangfang Cheng, Weifeng Yao, Beihua Bao, Kaicheng Zhang, Li Zhang, Anwei Ding

**Affiliations:** Jiangsu Collaborative Innovation Center of Chinese Medicinal Resources Industrialization, National and Local Collaborative Engineering Center of Chinese Medicinal Resources Industrialization and Formulae Innovative Medicine, Nanjing University of Chinese Medicine, Nanjing 210023, China; raindc@163.com (Y.C.); cff19870524@163.com (F.C.); njweifengyao@163.com (W.Y.); scotter01@163.com (B.B.); 20151501@njucm.edu.cn (K.Z.); zhangliguanxiong@163.com (L.Z.)

**Keywords:** pekinenin C, rat intestinal crypt epithelial cell line (IEC-6 cells), apoptotic mechanism, cell viability analysis, mitochondrial pathway, death receptor pathway

## Abstract

Pekinenin C is a casbane diterpenoid separated from the root of the traditional Chinese medicine, *Euphorbia pekinensis* Rupr., which is used as drug for the treatment of edema, ascites, and hydrothorax. Whereas pekinenin C exhibits severe cytotoxicity, the exact toxicity mechanism is unclear. In this study, the effects of pekinenin C on cell inhibition, cell cycle, and cell apoptosis were examined to explain its toxic mechanism. The proliferation of IEC-6 cells was accessed via MTT colorimetric assay after incubated with different concentrations of pekinenin C. Pekinenin C-treated IEC-6 cells labeled with RNase/PI and Annexin V/PI were analyzed by flow cytometric analyses for evaluation of cell cycle distribution and cell apoptosis, respectively. The apoptosis mechanism of pekinenin C on IEC-6 was investigated through assaying the activities of caspase-3, 8, 9 by enzyme-linked immunosorbent assay (ELISA), protein expression of Bax, Bcl-2, apoptosis-inducing factor (AIF), Apaf-1, Fas-associated death domain (FADD) and type 1-associated death domain (TRADD) by Western-blot, mRNA expression of Fas receptor (FasR), Fas ligand (FasL), tumor necrosis factor receptor (TNFR1) and NF-κB by RT-PCR. The results showed that pekinenin C has exhibited obvious IEC-6 cells toxicity and the IC_50_ value was 2.1 μg·mL^−1^. Typical apoptosis characteristics were observed under a transmission electron microscopy, and it was found that pekinenin C could cause G0/G1 phase arrest in IEC-6 cells in a dose-dependent manner and induce apoptosis of IEC-6 cells. Additionally, pekinenin C could increase the expressions of Bax, AIF, Apaf-1, FasR, FasL, TNFR1 and NF-κB, suppress the expression of Bcl-2, FADD and TRADD, then activate caspase-3, 8, 9 cascades, and at last result in apoptosis. These results demonstrated that pekinenin C effectively promoted cell apoptosis, and induced IEC-6 cells apoptosis through both the mitochondrial and death receptor pathways.

## 1. Introduction

Euphorbia Pekinensis Radix (EPR), the roots of *Euphorbia pekinensis* Rupr., which belongs to the Euphorbiaceae family with more than 2000 species, is characterized by the presence of milky latex [1]. As a toxic Chinese medicinal herb, Euphorbia pekinensis (*E. pekinensis*) has been used in China for thousands of years for the treatment of edema distention and fullness, hydrothorax and ascites, *etc*. [2]. However, the clinical application of *E. pekinensis* had some side effects because it might irritate skin, oral and gastrointestinal mucosa.

We assume that the toxicity of *E. pekinensis* may be related to a lot of diterpenoids [3,4,5,6,7]. Recent studies further showed that casbane diterpenoid exhibited more severe cytotoxicity than other diterpenoids [4,6,7]. 5α-hydroxyl-1βH,2αH-casba-3*Z*,7*E*,11*E*-trien-18-al, named pekinenin C (PC) was one member of casbane diterpenoid extracted from the petroleum ether fraction of *E. pekinensis*. It was shown that this compound could inhibit growth of many cancer cell lines. The C-5 hydroxy group and the double bond (Δ^11,12^) played important roles in the structure-activity relationships [7]. PC, a casbane diterpenoid separated from EPR, has been studied that it has high hepatocyte and gastro cytotoxicity against LO2 and GES-1 cell lines via MTT colorimetric assays [8]. However, its toxic mechanism is not clear. Therefore, in this work, the cytotoxicity of PC was estimated on rat intestinal crypt epithelial cell line (IEC-6 cells) and the effects of pekinenin C on cell inhibition, cell cycle, and cell apoptosis were investigated, which made an attempt to uncover the intestinal cytotoxic mechanism of EPR.

IEC-6 cells originated from crypt cells as judged by morphological and immunological criteria were chosen as a model cell line. It was described by Quaroni and colleagues as a homogenous population of epithelial-like cells with large, oval nuclei, and grows as tight colonies of polygonal, closely opposed cells. IEC-6 cells were non-transformed and retained the undifferentiated character of epithelial cells [9]. It was widely used in the study of intestinal epithelial cell growth [10,11], differentiation and metabolism [10,12].

There were two classic activation mechanisms of apoptosis: the death receptors pathway (extrinsic way) and the mitochondrial pathway (intrinsic way) [13,14,15]. The death receptors pathway was mediated through the cell-surface death receptors, such as tumor necrosis factor receptor (TNFR) and Fas, with caspase-8 acting as a key protease in this pathway [13,16]. Apoptosis induced by TNFR was associated with tumor necrosis factor receptor type 1-associated death domain (TRADD) recruitment while activation of Fas receptor (FasR) led to recruitment of Fas-associated death domain (FADD). For the mitochondria-mediated apoptotic pathway, the mitochondrial membrane potential decreases through electron leak pathway, and reactive oxygen species occurred in mitochondria, which opened mitochondrial permeability transition pore. As a result, a large number of pro- apoptotic proteins, including cytochrome C, apoptosis-inducing factor (AIF), apoptosis protease activating factor 1 (Apaf-1), are released from mitochondria into cytoplasm through this pore, triggering the activation of caspase-9, and subsequently the caspase cascade, leading to apoptosis [14].

Herein, the cytotoxicity of PC on IEC-6 cells was investigated by evaluating effects of PC on cell inhibition and morphology, demonstrating that PC had a high cytotoxicity against IEC-6 cells. Cell cycle and cell apoptosis treated with PC were confirmed by flow cytometry and mitochondrial ultrastructure, which showed that PC could arrest IEC-6 cells in G0/G1 phase in a concentration-dependent manner and result in apoptosis of IEC-6 cells. In addition, evidences indicated that PC induced apoptosis through the mitochondrial and death receptors pathways, in which the expression level of Bax, AIF, Apaf-1, and caspase-3, 8, 9 activity, FasR (Fas receptor), FasL (Fas ligand), NF-κB, and TNFR1 mRNA expression level were up-regulated and that of Bcl-2, FADD and TRADD protein down-regulated.

## 2. Results

### 2.1. Effects of Pekinenin C (PC) on Cell Viability Analysis

**Figure 1:**
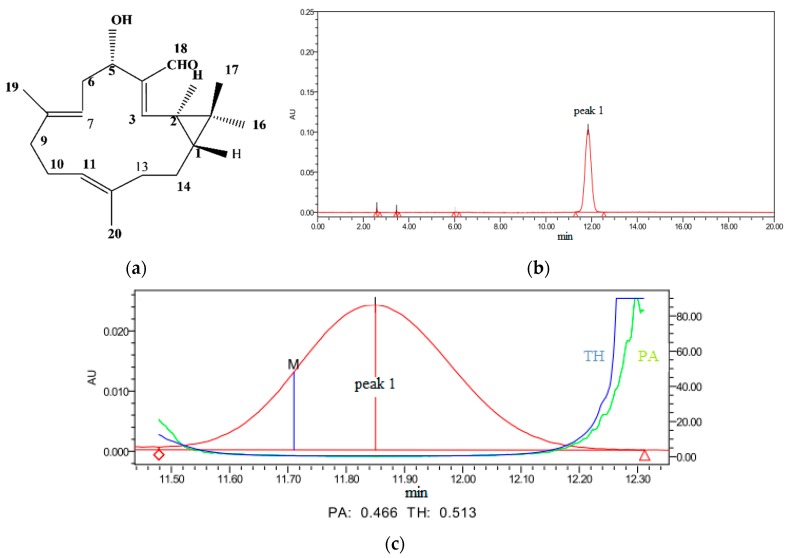
Structure of pekinenin C (PC) (**a**); the High Performance Liguid Chromatography (HPLC) chromatograms of PC (**b**) and the purity characterization of PC (**c**). TH is the abbreviation of threshold, PA is the abbreviation of purity angle and M is the abbreviation of point of maximum impurity.

PC (Figure 1a) is a diterpenoid separated from a traditional Chinese medicine plant EPR, also called JingDaji (Chinese name). It was prepared by preparative liquid chromatography and the purity had been determined by High Performance Liguid Chromatography (HPLC) to be 99% (Figure 1b,c).

**Figure 2:**
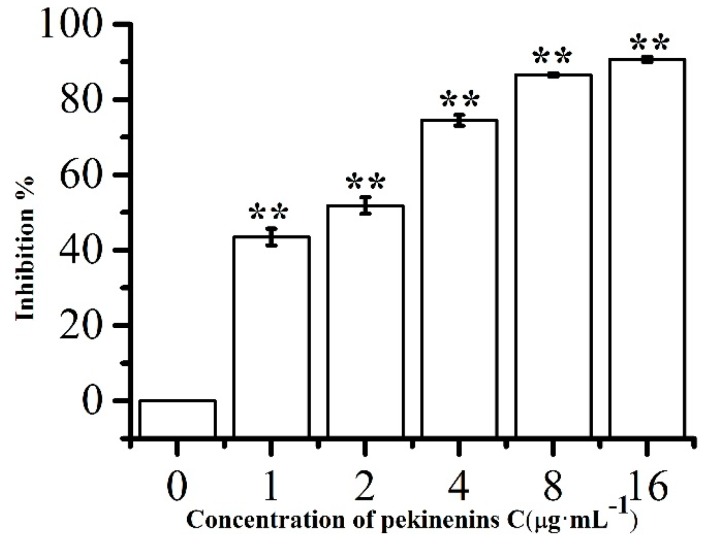
Relative cell viabilities of IEC-6 cells after incubation with various concentrations of PC. Compared with corresponding control group, ** *p* < 0.01, (*n* = 5).

Cytotoxicity was assayed by 3-(4,5-dimethylthiazol-2-yl)-2,5-diphenyltetrazolium bromide (MTT) method as previously described [17]. IEC-6 cells were cultured with increasing concentrations of PC, which were 1.0, 2.0, 4.0, 8.0 and 16.0 μg·mL^−1^ after 48 h exposure (Figure 2). Cell viability markedly decreased and the cell inhibition remarkably increased in a concentration-dependent manner (*p* < 0.01) compared with control group. The IC_50_ value of PC against IEC-6 cells were about 2.1 μg·mL^−1^ (approximately 6.95 μM). Therefore, in the subsequent studies, the concentrations of PC were set at 1.0, 2.0, 4.0 μg·mL^−1^ for cell apoptosis and cell cycle assay and 0.5, 1.0, 2.0 μg·mL^−1^ for caspase activity assay, and the expressions of Bax, Bcl-2, AIF, Apaf-1, FADD, TRADD FasR, FasL, TNFR1 and NF-κB mRNA expressions.

### 2.2. Effects of PC on Cell Cycle

**Figure 3:**
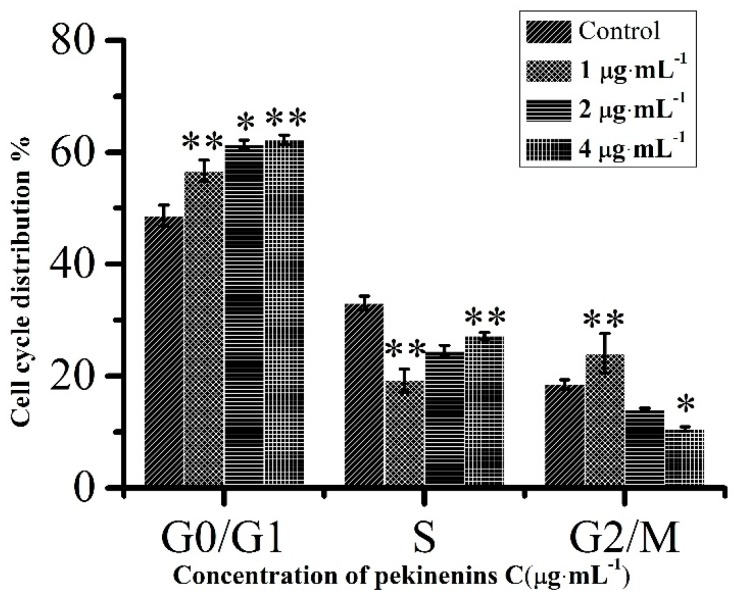
Cell cycle distribution of IEC-6 cells. Compared with corresponding control group, * *p* < 0.05, ** *p* < 0.01, (*n* = 3).

Cell cycle is usually regarded as a primary factor in cell proliferation, differentiation, migration and survival. It had been reported that G1-phase arrest could be significantly associated with apoptosis [18]. Through flow cytometry analysis, the percentage of G0/G1 phase cells increased from 56.70% to 62.24% after treatment with PC for 48 h (Figure 3), indicating PC arrested IEC-6 cells at G0/G1 phase in a concentration-dependent manner, blocked cell cycle progression, interfered DNA synthesis, and finally led to the apoptosis of IEC-6 cells.

### 2.3. Effects of PC on Cell Apoptosis

**Figure 4:**
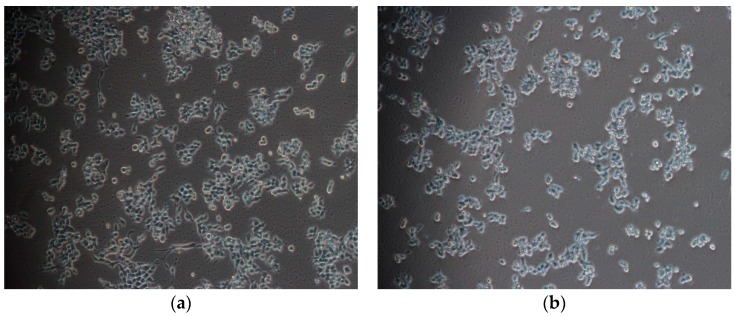

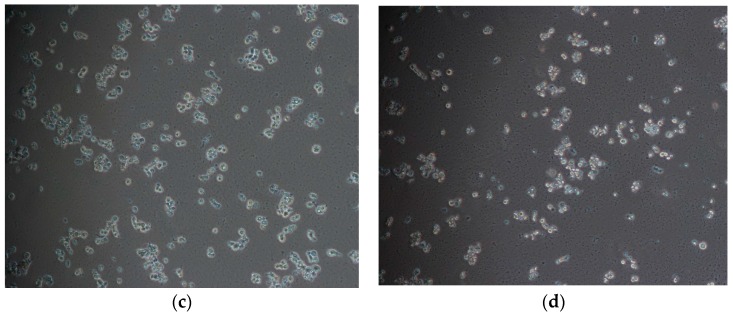
PC-induced inhibitory cell proliferation in IEC-6 cells were detected by inverted phase contrast microscopy (×200), the morphology of IEC-6 cells changed in the PC-treated groups. (**a**) Control; (**b**) 1 μg·mL^−1^; (**c**) 2 μg·mL^−1^; (**d**) 4 μg·mL^−1^.

Programmed cell-death (PCD) is death of a cell in any form, mediated by an intracellular program and apoptosis is the process of PCD [19]. After incubating with PC for 48 h, IEC-6 cells were observed with inverted phase contrast microscopy. Morphological changes of IEC-6 cells obviously occurred in the PC-treated groups (1.0, 2.0, 4.0 μg·mL^−1^ for 48 h) in contrast with the control group (Figure 4). The number of cells decreased, cell morphology changed from normal spindle to round, cell size became shrunken, cell skeleton arranged irregular, and cells began to lose the borders with surrounding cells. All these demonstrated that PC changed the cellular morphology and cell apoptosis appeared in IEC-6 cells.

**Figure 5:**
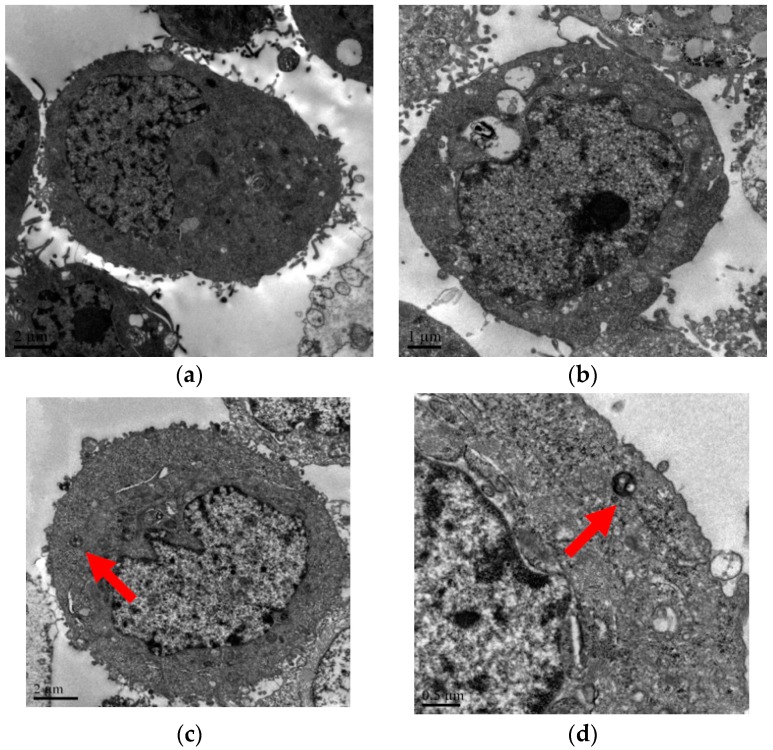
Transmission electron microscopy images of IEC-6 cell in control group (**a**) and treatment with PC of 2.0 µg·mL^−1^ (**b**–**d**) for 48 h; Cell morphology became more round shaped (**b**); red arrow in (**c**) showed nuclear chromatin condensation, red arrow in (**d**) showed formation of apoptotic body.

Results from transmission electron microscope (TEM) also indicated that apoptosis took place in the PC-treated group after 48 h, compared with the control group (Figure 5). In Figure 5a, cells shape were round and complete and intestinal villi were arranged regularly with abundant mitochondria in cytoplast. PC-treated cells appeared microscopic ultrastructure changes with apoptotic characteristics, for example cell morphology became more round shaped Figure 5b, nuclear chromatin condensated and aggregated Figure 5c, apoptotic body formed Figure 5d.

**Figure 6:**
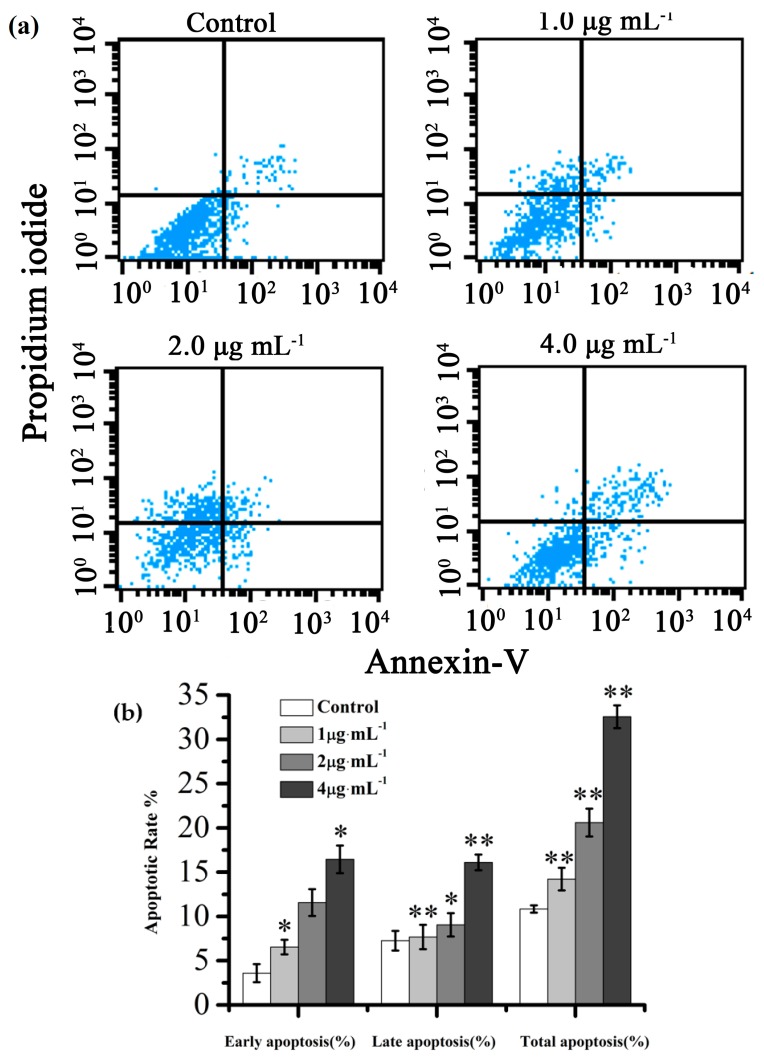
(**a**) Apoptosis assay using flow cytometer after Annexin V-FITC/PI staining; (**b**) Quantitative analysis of the number of apoptotic cells. Compared with corresponding control group, * *p* < 0.05, ** *p* < 0.01, (*n* = 3).

Annexin V-FITC/PI dual staining were analyzed to investigate the apoptosis effect of PC on IEC-6 cells *in vitro*. As shown in Figure 6, when the concentration changed from 1.0 to 4.0 μg·mL^−1^ after 48 h PC-treated, the proportion of total apoptotic cells significantly increased from 20.83% to 37.91%. However, the percentages of apoptotic cells compared with the control was only 10.84%. The fluorescence intensity of FITC and PI in IEC-6 cells treated with PC at high doses of 4.0 μg·mL^−1^ was noticeably enhanced, which revealed that high dose PC caused obvious cell apoptosis, in accordance with the Annexin V-FITC/PI dual staining assay.

### 2.4. Effects of PC on Caspase-Dependent Mitochondria Pathway

**Figure 7:**
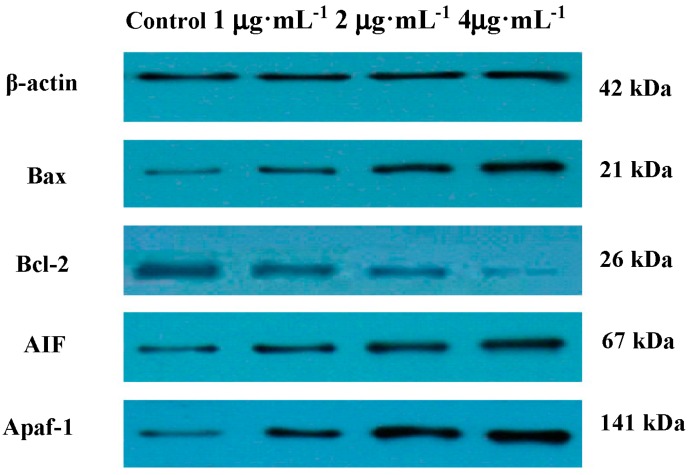
The expression of Bax, Bcl-2, AIF, Apaf-1 protein detected by western-blotting after treatment with PC for 48 h.

The Bcl-2 family is the main regulatory factors in the process of mitochondrial pathway apoptosis. The family members comprise anti-apoptotic (Bcl-2, Bcl-w, Bcl-XL, and Mcl-1) and pro-apoptotic members (Bax, Bak, Bim, Bad, Bid, Puma, and DP5) [20,21]. Some Bcl-2 family proteins can activate caspase-9 and caspase-3 leading to the execution phase of cell apoptosis [22,23]. Moreover, Bcl-2 is reported to be able to suppress the AIF pathway [24]. AIF involved in inducing a caspase-independent pathway of apoptosis. Western blot analysis showed an increase in expression of pro-apoptotic protein Bax, AIF and Apfa-1, and a decrease in expression of anti-apoptotic protein Bcl-2 (Figure 7).

**Figure 8:**
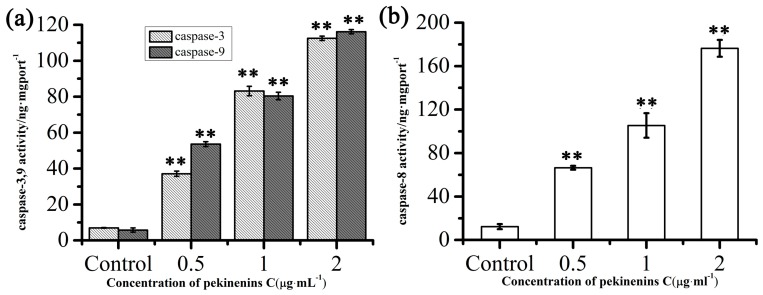
Effects of PC on IEC-6 cells caspase-3, 9 (**a**) and caspase-8 (**b**) activity after treatment for 48 h. Compared with corresponding control group, ** *p* < 0.01, (*n* = 3).

The interaction of apoptosis protease activating Apfa-1 and caspase-9 results in the formation of apoptosome, which activated caspase-9, leading to caspase-3 and induction of apoptosis. To further understand the apoptosis signaling induced by PC, the activation of caspase cascades was assessed. After PC treatment for 48 h, which was chosen from 12, 24 and 48 h, the activation of caspase-3, 9 was detected with enzyme-linked immunosorbent assay (ELISA) kit. The results suggested that the activation of caspase-3, 9 in IEC-6 cells were obviously elevated by PC in a concentration-dependent manner (Figure 8a). Results demonstrate that PC promoted IEC-6 cells via the mitochondrial apoptosis pathway.

### 2.5. Effects of PC on Death Receptor Pathway

**Figure 9:**
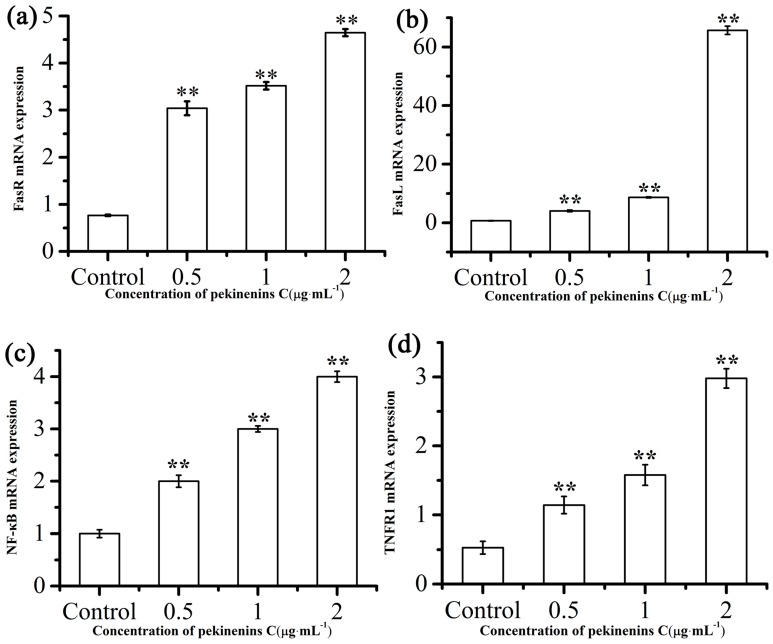
Effects of PC on FasR (**a**); FasL (**b**); TNFR1 (**c**) and NF-κB (**d**) mRNA expression level after treatment for 48 h. Compared with corresponding control group, ** *p* < 0.01, (*n* = 3).

The extrinsic death receptors pathway is another apoptosis signaling pathway. It might be mediated through the death receptors existed on the cell surface [25]. In this apoptotic process, apoptosis was mainly stimulated by drugs via death receptors, such as FasR, TNFR, DR3, DR4, DR5. To investigate whether PC in induced apoptosis in IEC-6 cells via a death receptors pathway, ELISA kit and RT-PCR were used to determine caspase-8 and FasR, FasL, TNFR and NF-κB mRNA expressions, respectively. We then determined TRADD and FADD if these changes in mRNA levels were accompanied by corresponding changes in protein levels. As Figure 8b, Figure 9 and Figure S1 showed, extrinsic pathway-associated genes, cleaved caspase-8, FasR, FasL, TNFR and NF-κB mRNA were up-regulated and FADD, TRADD were down-regulated in a concentration-dependent manner. These results indicated that PC involved in induction of apoptosis is also mediated by the death receptor pathway.

## 3. Discussion

In order to study the apoptosis mechanism of PC, PC-treated on cell inhibition, cell cycle, and cell apoptosis were investigated. MTT was a widely used regent to assess cell number because a yellow tetrazole is reduced to purple formazan in living cells and dimethyl sulfoxide (DMSO) is added to dissolve the insoluble purple formazan product into a colored solution. The absorbance of colored solution is quantified by measuring at 490 nm, which is proportional to the total of cells [26]. MTT assays showed PC could effectively improve cell inhibition. In addition, apoptosis was described by a lot of characteristic cell changes, such as cell shrinkage, nuclear fragmentation, chromatin condensation, chromosomal DNA fragmentation and the formation of apoptotic bodies [27]. In this work, the cellular morphology alterations including cells became more round and smaller (Figure 4), compared to control cells and characteristic features of apoptosis, including cell volume decreased, nuclear chromatin condensated and apoptotic body formed (Figure 5), could also be observed in PC treated cells.

Cell cycle is the series of events that involve G0 phase, G1 phase, S phase, G2 phase, and M phase [28]. In G0 phase, the cells are both quiescent and senescent, and have stopped dividing. Cells size increases in G1 phase and DNA replication occurs during S phase. The cells will continue to grow in G2 phase, then cell growth stops at the M phase and cellular energy is focused on dividing into two daughter cells [29]. In current study, the result indicated that PC could induce G0/G1 cell cycle arrest in a concentration-dependent manner. This conclusion was also consistent with flow cytometry analysis on cell apoptosis, which the percentage of total apoptotic cells revealed that PC notably promoted cell apoptosis of IEC-6 treated with PC for 48 h in a concentration-dependent manner.

Apoptosis can be initiated through two distinct signaling pathways [30,31]. The extrinsic pathway of apoptosis was referred to cell death induced by external factors such as FasL, TNF-α, and TNF-related apoptosis-inducing ligand (TRAIL) that activate caspase-8, which in turn activate downstream effector caspases such as caspase-3 [32,33]. The intrinsic apoptotic pathway was characterized by permeabilization of the mitochondria via Bcl-2 family proteins and activation of the caspase family [34], which included caspase-3, and caspase-9, *etc*. As shown in Figure 8, PC could effectively elevated the activation of caspase-3, 8, 9, which was likely to demonstrate that PC-induced apoptosis between death receptors and mitochondrial-associated pathways.

Bcl-2 family of proteins is known as an important gatekeeper to the apoptotic pathway. As an anti-apoptotic member of Bcl-2 family, Bcl-2 was found primarily in the mitochondrial membrane, the nuclear membrane and the endoplasmic reticulum, and apoptotic resistance was significantly higher than in other sites [35]. As a pro-apoptotic member of Bcl-2 family, Bax existed in the cytoplasm as a monomer. Once stimulated by related apoptotic signals, Bax was transferred into the membrane of mitochondria, released from mitochondria into cytosol, and subsequently induced apoptosis [36]. In this work, treatment of IEC-6 cells with PC increased expression levels of Bax, Apaf-1 and AIF while Bcl-2 was decreased. Hence, from these results we might conclude activation of a mitochondrial-dependent pathway (Figure 7).

The death receptor pathway is mediated through the death-domain-containing receptors such as TNFR, FasR, death receptor 3 (DR3), death receptor 4 (DR4), death receptor 5 (DR5) [37]. Fas/FasL is the most representative pathway of apoptosis mediated by death receptors. Fas and TNFR1 belonged to type I transmembrane protein [38,39], and FasL (natural Fas ligand) was a type II transmembrane protein. As an integral membrane protein, TNFR1 can activate caspase-8. TNF-α signaling can stimulate the generation of an apoptotic signal via FADD-mediated activation of caspase-8, whereas TRADD activation can lead to activation of NF-κB and trigger downstream events that result in increased gene expression leading to an anti-apoptotic response [40]. Then the other members of the caspases family, such as caspase-3 were activated and ultimately apoptosis occurred within several hours [41]. NF-κB is a key protein complex in controlling transcription of DNA and regulating the immune response to infection [42]. The present study showed that FasR, FasL, TNFR and NF-κB mRNA levels could be enhanced by the treatment of PC, which provided further evidence that PC might act on death receptors pathway to induce apoptosis of IEC-6 cells.

## 4. Experimental Section

### 4.1. Plant Materials and the Preparation of PC

The roots of *Euphorbia Pekinensis* Rupr. were purchased from the Bozhou medicine market (Bozhou, China), and identified by Professor Wei Yue (Nanjing University of Chinese Medicine, Nanjing, China). PC was separated from the EtOAc fractions of *Euphorbia Pekinensis* Rupr. as reported previously [7]. PC was dissolved in DMSO at a concentration of 12.5 mg·mL^−1^ and the stock solution was then diluted with Dulbecco’s modified Eagle’s medium (DMEM) medium prior to use to obtain the indicated concentration.

### 4.2. Chemicals and Regents

DMEM and fetal bovine serum (FBS) were purchased from Gibco (Grand Island, NY, USA). MTT, DMSO and phosphate-buffered saline (PBS) were purchased from Beijing Solarbio science and technology Co., Ltd. (Beijing, China). Annexin V-FITC/PI apoptosis detection kit was purchased from Beyotime Biotechnology Co., Ltd. (Nantong, China). Caspase-3, 8, 9 Elisa kits were purchased from Nanjing Jin Yibai Biological Technology Co., Ltd. (Nanjing, China). Total protein extraction kit, Bradford protein assay kit and all antibodies were purchased from Nanjing Saiyan Bioengineering Institute (Nanjing, China).

### 4.3. Cell Culture

The rat intestinal crypt epithelial cell line (IEC-6 cells) which was purchased from the American Type Culture Collection (ATCC, Rockefeller, MD, USA), was cultured in DMEM containing 10% (*v*/*v*) FBS and 1% bovine insulin, 100 unit·mL^−1^ of penicillin and streptomycin at 37 °C in a humidified 5% CO_2_ incubator.

### 4.4. Cytotoxicity Assay

Cytotoxicity was assayed by the MTT assay as previously reported [16]. In brief, cells were diluted in serum-free DMEM medium and seeded in 96-well plates (1.0 × 10^4^ cells·well^−1^), then incubated at 37 °C for 24 h in a humidified atmosphere of 5% CO_2_ with 100 μL.

Every solution was added into 96-well plates with 100 μL per well. After 24 h, the growth medium was replaced with different concentration of PC (the final concentrations of 1.0, 2.0, 4.0, 8.0, 16.0 μg·mL^−1^) and the cells were incubated at 37 °C for 48 h in an incubator. Then, the treated cells were incubated with 20 μL per well of MTT (5.0 mg·mL^−1^) for a successive 4 h. The MTT-formazan product dissolved in 150 μL DMSO and the absorbance at 490 nm value of each well was measured by a Microplate Reader (Olympus, Tokyo, Japan). All the doses were tested at least five times. The inhibition rate of cell proliferation was calculated as: cell inhibition (%) = (1 − sample solution absorbance value/control absorbance value) × 100%.

### 4.5. Cell Cycle Analysis

Cells were seeded in 6-well plates (1.6 × 10^5^cells·well^−1^) and incubated at 37 °C for 24 h in a humidified atmosphere of 5% CO_2_. The growth medium was replaced with PC at doses of 1.0, 2.0, 4.0 μg·mL^−1^. After incubated for 48 h, cells were collected and placed in the centrifuge tube at 1500 rpm for 5 min and fixed in cooled 70% ethanol overnight at 4 °C. Cells were washed twice with PBS and stained with PI/RNase staining solution (Sigma, St. Louis, MO, USA) which kept in dark place for 30 min and analyzed by flow cytometer (FACSCalibur, BD Instruments Inc., Franklin Lakes, NJ, USA). Each experiment was performed triplicate.

### 4.6. Cell Morphological Detection and Transmission Electron Microscopy (TEM)

Cell pretreatment methods were the same with cell cycle analysis. After incubated for 48 h, cells were observed with phase contrast microscope (Olympus, Tokyo, Japan).

Cells were seeded in cell bottle at a density of 1.0 × 10^5^ cells·mL^−1^ in 5 mL medium solution and incubated at 37 °C for 24 h in a humidified atmosphere of 5% CO_2_. After being exposed to 2.0 μg·mL^−1^ PC for 48 h, fixed at room temperature in 2.5% glutaraldehyde buttered with 1% osmic acid for 1 h. The cells were then dehydrated with acetone and embedded in a mixture of Epon-812 araldite. The ultrathin sections were prepared with a microtome (Leica, Nussloch, Germany) and mounted on copper grids. The samples were detected in a transmission electron microscope (JEOL Ltd., Tokyo, Japan).

### 4.7. Western Blot Analysis

After the treatment of IEC-6 cells, protein samples were separated using 12% sodium dodecyl sulfate-polyacrylamide gel electrophoresis and transferred onto nitrocellulose membranes. After membranes were blocked in 5% BSA for 2 h, immunoblotting was conducted by incubating with the primary antibodies overnight at 4 °C. In this study, β-Actin (13E5) Rabbit mAb (42 kDa) antibodies and TRADD antibody were purchased from Cell Signaling (Boston, MA, USA), Anti-FADD antibody (ab24533) were purchased by ABCAM (Shanghai, China). After incubation with goat anti rabbit IgG-HRP (SN134, Nanjing Sunshine Biotechnology Co., Ltd., Nanjing, China) secondary antibodies for 2 h at room temperature, immunoreactive bands were visualized and analyzed using a chemical luminescence (ECL) substrate (Millipore, Darmstadt, Germany) and automatic chemical luminescence (ECL)/fluorescence image analysis system named Gel Image System (Tanon Science & Technology Co., Ltd., Shanghai, China).

### 4.8. Caspase-3, 8, 9 Activation Assay

The pretreatment of cells were the same with cell cycle analysis, then were treated with PC at various concentration of 0.5, 1.0, 2.0 μg·mL^−1^. After 48 h, cells and the supernatants were obtained for measurement of caspase-3, 8, 9 activation according to the manufacturer’s instructions. The activation of caspase-3, 8, 9 were measured spectrophotometrically at a wavelength of 450 nm within 15 min. The concentration of caspase-3, 8, 9 was then determined by comparing the optical density (OD) of PC-treated to the standard curve.

### 4.9. RNA Isolation and Real Time Quantitative PCR

Total RNA was isolated from treated IEC-6 cells using Trizol reagent (Sigma, St. Louis, MO, USA) following the protocol provided by the manufacturer. Glyceraldehyde phosphate dehydrogenase (GAPDH) was used as the invariant control. After the treatment, cells were washed twice with ice-cold PBS and then were used for the fluorescence real-time PCR detection (Nanjing Saiyan Bioengineering Institute, Nanjing, China). The relative expression of RNA was calculated using 2^−ΔΔ*C*t^ method [43].

### 4.10. Statistical Analysis

The data were analyzed with software SPSS 15.0 (SPSS Inc., Chicago, IL, USA, 2008) and presented as mean values ± SD. The results were analyzed using one-way analysis of variance (ANOVA) and an unpaired student *t*-test. Statistical significance was indicated as values of *p* < 0.05.

## 5. Conclusions

In conclusion, the current study highlighted the apoptotic mechanism of PC in IEC-6 cells. PC exerted serious intestinal cell toxicity by inducing apoptosis and cell cycle arrest. The data indicated the key role of Bcl-2/Bax in mitochondrial mediated apoptosis by activating caspase-3. PC induced caspase-dependent apoptosis, which is connected with caspase-9 and other members of the Bcl-2 family. Together with the death receptors pathway, FADD and TRADD protein levels were consistent with mRNA expression. All these results support the apoptotic mechanism of PC can be activated both the death receptors pathway and the mitochondrial-dependent pathway.

## Supplementary Materials

Supplementary materials can be found at http://www.mdpi.com/1422-0067/17/6/850/s1.
