# Supplementary material for: Toxicity of Pekinenin C from Euphorbia Pekinensis Radix on Rat Small Intestinal Crypt Epithelial Cell and Its Apoptotic Mechanism
Source: Int J Mol Sci. 2016 Jun 2;17(6):850. doi: 10.3390/ijms17060850 (PMC4926384; doi:10.3390/ijms17060850)
Supplement: Supplementary file 1 [file ijms-17-00850-s001.pdf]

Supplementary Materials: Toxicity of Pekinenin C from Euphorbia Pekinensis Radix on Rat Small Intestinal Crypt Epithelial Cell and Its Apoptotic Mechanism

Yudan Cao, Fangfang Cheng, Weifeng Yao, Beihua Bao, Kaicheng Zhang, Li Zhang and Anwei Ding

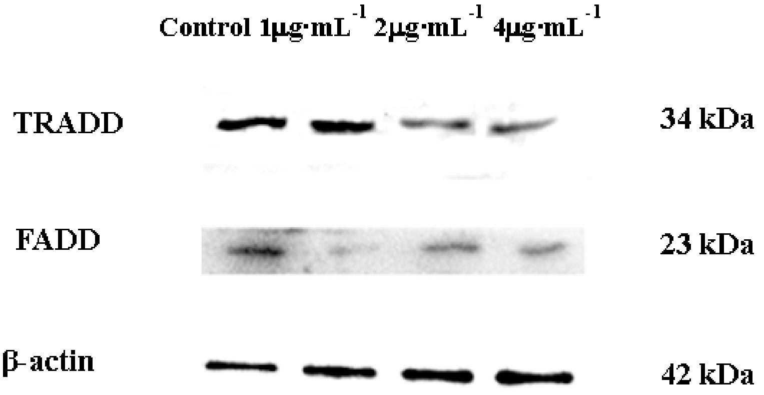

Figure S1. The expression of TRADD, FADD protein detected by western-blotting after treatment with pekinenin C (PC) for 48 h.
